# Development of a Community Commitment Scale with Cross-sectional Survey Validation for Preventing Social Isolation in Older Japanese People

**DOI:** 10.1186/1471-2458-12-903

**Published:** 2012-10-24

**Authors:** Ayumi Kono, Etsuko Tadaka, Yukiko Kanaya, Yuka Dai, Waka Itoi, Yuki Imamatsu

**Affiliations:** 1 School of Nursing, Osaka City University, 1-5-17 Asahi, Abeno, Osaka, 545-0051, Japan; 2 School of Nursing, Yokohama City University, 3-9 Fukuura, Kanazawa, Yokohama, Kanagawa, 236-0004, Japan

**Keywords:** Community commitment, Elderly social isolation, Neighbor, Scale development, Self-efficacy for helping elderly neighbors

## Abstract

**Background:**

Elderly social isolation could be prevented by facilitating communication or mutual helping at the neighborhood level. The helping of elderly neighbors by local volunteers may relate to their community commitment (CC), but ways to measure CC have not been identified. The aim of the present study was to develop a Community Commitment Scale (CCS) to measure psychological sense of belonging and socializing in the community among local volunteers, for research in prevention of elderly social isolation. We also tested the CCS in a general population of the elderly.

**Methods:**

A pilot test of 266 Japanese urban residents was conducted to examine face validity for 24 identified items, of which 12 items were selected for the CCS, based on a 4-point Likert-type scale. The CCS was developed via self-report questionnaires to 859 local volunteers in two suburban cities and to 3484 randomly sampled general residents aged 55 years or older living in one of the cities. To assess concurrent validity, data were collected using the Brief Sense of Community Scale (Peterson; 2008) and two types of single questions on self-efficacy for helping elderly neighbors.

**Results:**

Item analysis and factor analysis identified 8 items, which were classified between two datasets under the domains of “belonging” and “socializing” in the local volunteers and the general residents. Cronbach’s alpha (which conveyed the internal consistency of the CCS) was 0.75 in local volunteers and 0.78 in general residents. The correlation coefficients between the scores of the CCS and BSCS were 0.54 for local volunteers and 0.62 for general residents. ANOVA comparing the CCS between the confidence levels of the two types of single question of self-efficacy on helping elderly neighbors showed a strong relationship in the volunteers and residents.

**Conclusions:**

These results demonstrate acceptable internal consistency and concurrent validity for the CCS, with the two dimensions “belonging” and “socializing”, among the local volunteers and general residents in urban Japanese areas. Community commitment measured by the CCS was related to the degree of confidence for self-efficacy in helping elderly neighbors to prevent elderly social isolation.

## Background

Social isolation, defined as extremely limited social support, contributes to higher risks of disability, poor recovery from illness, and early death in older people [1]. Socially isolated older people are the most vulnerable to natural disasters [1-3]. Particularly in Japan, “solitary death” means passing away at home and unnoticed by anyone, with bodies left unattended for several days or even over months or years [4], with estimates of the incidence rate at 0.10 per 1000 persons [5].

Preventing social isolation in older people has become a high priority for public health policy in the present “super-aging” society, where people aged 65 years or over constituted 23.1% of the population in 2010 [6]. It is suggested that one of the public health strategies for preventing elderly social isolation is to facilitate mutual natural helping at the neighborhood level, where the numbers of older people living alone are increasing and local community cohesion is collapsing [7].

There have been some trials [8-10] to enhance mentoring by local volunteers for prevention of social isolation in older people. Most Japanese local governments have unique informal community-based organizations or systems, composed of local volunteers (including residents in neighborhood associations, district welfare commissions, or volunteers) who support elders living in the districts with municipal community-based comprehensive care centers [11]. According to US reports [12,13], the support of elderly neighbors by local volunteers may be facilitated by the community commitment (CC) of the volunteers, defined as psychological sense of belonging and socializing in community. However, this relationship between helping elderly neighbors and CC has not been demonstrated in Japanese local volunteers.

A concept similar to CC is Sense of Community (SOC), representing a broader concept of community and people’s relation to it compared to CC [14]. An SOC model which specifies four dimensions, including fulfillment, group membership, influence, and emotional connection [15,16], has been developed in community psychology disciplines for decades. Previous studies have shown that a high level of SOC related to favorable health measures [17,18], social support, or well-being [19-21] among residents in the community. Several SOC measures have been developed and their utility or validity tested [16,22-24], mostly in western countries.

Because SOC could differ by ethnic groups [25,26], it is important to account for cultural diversity while measuring the community psychological aspects of residents [27]. Particularly because Japanese people tend to value neighborhood connection more than people in western countries, Ishimori suggested that community sense or commitment should be developed in the Japanese context [28]. In 1978, the Attitude toward Community Scale for Japanese general residents was developed [29] and it has recently been modified to include wording for use in the present modern Japanese society and has shown an association with health-related outcomes in general residents [30]. Those scales have focused on measuring general attitudes to the local community, including an item which indicates helping the elderly living alone (item 8) [30]. We hypothesized that there is a distinct CC which correlates with the tendency to help elderly neighbors and that this could be an outcome indicator for evaluating community-based intervention by local volunteers, including helping elderly neighbors to reduce social isolation. However, whether CC relates to the helping of elderly neighbors by local volunteers in Japan has not been clarified, nor have ways to measure these even been identified.

The aim of the present study was to develop and validate a community commitment scale (CCS) to measure psychological sense of belonging and socializing among local volunteers, for prevention of social isolation among older people in Japan. We also tested the CCS in general residents to confirm the composition of items, the internal consistency, and the concurrent validity, because local volunteers are selected from among general residents, even though we have intended to focus the use of the CCS on local volunteers.

The present scale development process included; 1) assessment of the face validity to identify the tentative items for the CCS through a pilot test, 2) refinement of the items of the CCS using a larger group of local volunteers as target subjects, and a cross-section of general residents aged 55 years or over as control subjects, 3) demonstration of internal consistency, and 4) assessment of concurrent validity using the Brief Sense of Community Scale (BSCS) [23] and two types of a single question on self-efficacy for the natural helping elderly neighbors.

## Methods

### Pilot test

#### Generating the item pool and assessing the face validity

We generated an item pool (24 items) to measure CC using literature reviews [16,22-24,29,30] or our own raw interview data from previous study projects [31], that consisted of three dimensions: “concerns for the elderly” (8 items); “belonging and contribution”(8 items); and “cohesion and socializing” (8 items), to be tested with 4-point Likert-type scaling. The item pool was reviewed by 8 knowledgeable experts, including community health nurses or social workers, to check the face validity including the items’ clarity and proper reading level [32] and the 24 initial items were refined.

#### Procedure of the pilot test

A total of 297 local volunteers (100.0%) were invited to take part in the pilot test at community meetings in two urban districts in Japan. Because the CCS could be adapted to local volunteers, we surveyed a group of them for the pilot test. Participants were the 266 volunteers who responded (89.6%). Their mean age was 67.2 (SD 1.7) years old; 163 persons (61.5%) were female; and the mean years living in the area were 34.2 (16.0) years.

Data including the 24 initial items of the CCS were collected via self-administered written questionnaire at meetings from November 2009 to January 2010. Each item was scored 0 (strongly disagree), 1 (slightly disagree), 2 (slightly agree), or 3 (strongly agree). We included 8 negatively worded items, and set the response of “I can’t understand” in each item to check the difficulty of response.

#### Item analysis

Item analysis included assessment of the response difficulty, distribution, and item-to-total correlation (see Additional file 1: Appendix: Table S1).

First, we assessed the rates of response difficulty including missing data or data with responses of “I can’t understand”, which of all items were less than 5.0%, so that we did not omit any items on that basis. Second, assessing rates of distribution of responses, 5 items were omitted because the frequency of responses of “slightly agree” and “strongly agree” were about 95%. Finally, after we investigated item-to-total correlation, we excluded those same 5 items as well. Another 5 items were omitted because the correlation coefficients of each were under 0.35 [31]. Thus, 2 items of “concerns for the elderly”, 5 items of “belonging and contribution”, and 7 items of “cohesion and socializing” remained. Because 2 items are too few to compose a dimension of “concerns for the elderly”, we excluded them.

#### Factor analysis

The promax rotation estimating two factors was conducted for factor analysis with the 12 retained items. The contribution of the factor named “socializing” (4 items) was 0.38 and that of “belonging” (8 items) was 0.13, for a cumulative contribution of 0.51 (see Additional file 1: Appendix: Table S2). We decided to investigate these tentative 12 items of the CCS with a further survey. Responses of “I can’t understand” in each item were treated as missing values in the analysis.

### Settings

The main survey was performed from October 2010 to March 2011 at three suburban cities, Daito and Matsubara in Osaka and Yokohama in Kanagawa, Japan. The population of Daito in 2010 was 124,275, that of Matsubara was 124,398, and that of Yokohama was 3,627,000. Daito and Matsubara are close to central Osaka, and Yokohama is the capital of Kanagawa prefecture and includes suburban and urban areas.

### Participants and procedure

**Figure 1:**
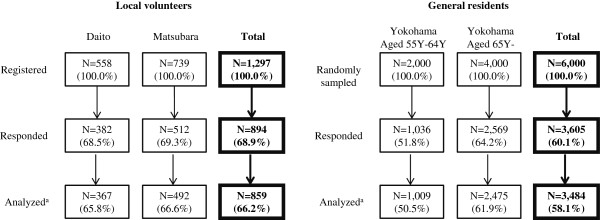
**Selection of the study of participants. **^**a**^**Excluding the data missing gender or age.**

Selection of the study participants is shown in Figure 1.

For the target subjects, all of the local volunteers who registered at two local governments (Daito and Matsubara) were recruited at their community meetings and questionnaires were delivered. Registered local volunteers who engaged in activities for helping community-dwelling elders in Daito were 558 people (100%) and those in Matsubara were 739 people (100%). Responding participants in Daito were 382 people (68.5%) and those in Matsubara were 512 people (69.3%). Analyzed participants in Daito were 367 people (65.8%) and those in Matsubara were 492 people (66.2%), since we excluded the data missing gender or age. Finally, 859 local volunteers (66.2%) were left for the analysis.

For the general subjects, 2,000 residents from the population aged above 55 years and less than 64 years, and 4,000 residents aged 65years and older were randomly sampled from the population on the residential enrollment list of Yokohama. We selected residents aged 55 years and older, because the ages of most local volunteers were in this age range. From the extracted sample of 6,000 residents (100.0%) to whom questionnaires were mailed, 3,605 people (60.1%) responded and 3,484 general residents (58.1%) were left for the analysis. Data were collected via anonymous postal self-administrated written questionnaire in both surveys.

### Measures

The tentative 12 items of the CCS included “belonging” (Q1-Q8) and “socializing” (Q9-Q12). Each item of the CCS was scored from 0 to 3, including in the pilot study. We translated the CCS from Japanese to English carefully, via back translation by a bilingual researcher specializing in qualitative research, to publish the CCS in English. The face validity of the CCS English version was also reviewed by a researcher in community health nursing in the United States.

To assess the concurrent validity, we investigated two aspects. One aspect is traditional SOC measured by the BSCS developed in the United States [23,24] based on the theory of McMillan and Chavis [15]. The BSCS includes 8 items scored from 0 for “strongly disagree” to 4 for “strongly agree”, providing a range of 0 to 32. High scores by the BSCS indicate a high level of SOC. Since the BSCS had never been used for any surveys in Japan, we conducted a back translation for the BSCS in the same manner as for the CCS for the present study. Regarding the internal consistency of the BSCS, Cronbach’s alphas were 0.90 and the face validity of the BSCS was checked by community health nurses.

Another aspect was the use of self-efficacy for helping elderly neighbors to clarify the relationships with CC. We hypothesized two types of activities of helping elderly neighbors to prevent elderly social isolation: “I can call on elderly neighbors, if I do not see them for a few days” and “I can help elderly neighbors with grocery shopping or garbage disposal chores”. Participants were asked their degree of confidence (including “not confident at all”, “not confident slightly”, “slightly confident”, or “absolutely confident”) regarding the activities.

### Ethical approval

We informed potential participants about the survey via mail for the general residents and via oral explanation and documents for the local volunteers, including the pilot study. The present study protocol was approved by the Institutional Review Board of Yokohama City University (No. A111124011).

### Statistical analysis

Analysis was done using SAS version 9.2 statistical software and consisted of the following: 1) calculating the distribution of responses of “slightly agree” plus “strongly agree”, the skewness, and the kurtosis of each item of the CCS to show the normality of the variables for the application of parametric analysis; 2) the promax rotation for the factor analysis to refine the items; 3) calculating Cronbach’s alpha to examine internal consistency; 4) calculating the Pearson’s correlation coefficients between the CCS scores and the BSCS scores; and 5) calculating eta squares for effect size by analysis of variance to compare the CCS scores between confidence levels of the two questions of self-efficacy on helping elderly neighbors.

## Results

### Characteristics of study participants

Characteristics of the participants are shown in Table 1. Of the local volunteers, 60.9% of them were female, 73.9% of them aged from 60 to 75 years, and 86.4% of them lived in the area more than 30 years. Of the general residents, 51.7% of them were female, 61.3% of them aged from 60 to 75 years, and 52.3% of them lived in the area more than 30 years.

**Table 1:** Characteristics of Study Participants

	**N(%)**	**Local volunteers 859(100.0)**	**General residents 3,484(100.0)**
Gender, N (%)	Female	523 (60.9)	1,800 (51.7)
Age, N (%)	−55y	70 (8.2)	
	55-60y	75 (8.7)	419 (12.0)
	60-65y	157 (18.3)	590 (16.9)
	65-70y	236 (27.5)	829 (23.8)
	70-75y	241 (28.1)	716 (20.6)
	75-80y	59 (6.9)	524 (15.0)
	80-85y	17 (2.0)	276 (7.9)
	85-90y	4 (0.5)	95 (2.7)
	90-95y		32 (0.9)
	95-		3 (0.1)
Living arrangement, N (%)	Living alone	75 (8.8)	431 (12.6)
	Couple	369 (43.1)	1451 (42.3)
	Living with children	394 (46.0)	1430 (41.7)
	Other	18 (2.1)	122 (3.6)
Years of living in the area, N (%)	−10y	22 (2.6)	393 (11.3)
	10-20y	29 (3.4)	540 (15.6)
	20-20y	66 (7.7)	721 (20.8)
	30y-	742 (86.4)	1816 (52.3)
Born in the city, N (%)	Yes	226 (26.4)	915 (26.4)
Owning house, N (%)		784 (91.4)	2929 (85.1)
Having job, N (%)	Yes	232 (27.2)	1199 (35.0)

### Distribution in each item of the CCS

When the distribution of each item of the CCS was examined, those of the general residents were normal. However, in the local volunteers, we found that the distribution was 95.3% and the skewness was 1.03 for item Q1, and that the distribution was 93.6%, the skewness was 1.30, and the kurtosis was 1.52 for item Q2 (see Additional file 1: Appendix: Table S3). Because the CCS was being developed for local volunteers, these items, Q1 and Q2 in “belonging”, were excluded from the factor analysis.

### Factor analysis

The results of factor analysis in the local volunteers and general residents (see Table 2) show that item composition was the same, that is, one factor named “socializing” was composed of 6 items (Q5, Q6, Q9-Q12) and another factor named “belonging” was composed of 4 items (Q3,Q4, Q7, Q8).

**Table 2:** Factor analysis in local volunteers and general residents

		**Local volunteers N=859**			**General residents N=3,484**		
		**Factor loading**		**Communality**	**Factor loading**		**Communality**
		**I**	**II**		**I**	**II**	
*Socializing*							
Q10	My neighbors speak regularly and are concerned for one another.	0.81	0.14	0.67	0.82	0.22	0.69
Q9	My neighbors often greet one another.	0.72	0.01	0.57	0.71	0.15	0.52
Q11	I enjoy spending time with my neighbors.	0.79	0.41	0.66	0.83	0.40	0.70
Q12	My neighbors help me whenever I am in need.	0.73	0.25	0.54	0.79	0.26	0.62
Q6	The neighborhood association activities foster friendships among local residents.	0.67	0.48	0.53	0.70	0.36	0.50
Q5	I feel that my neighborhood association activities are worth doing.	0.56	0.51	0.44	0.65	0.42	0.46
*Belonging*							
Q3^a^	I don’t feel I am a member of this community.	0.15	0.70	0.50	0.13	0.55	0.31
Q7^a^	Socializing in my community is annoying and complicated.	0.34	0.75	0.57	0.40	0.80	0.65
Q8^a^	I am not interested in my neighbors.	0.14	0.67	0.46	0.31	0.76	0.58
Q4^a^	I am hesitant to take part in my neighborhood association activities, because my duties might increase.	0.21	0.69	0.48	0.22	0.69	0.48
Contribution, %		0.37	0.19		0.40	0.15	
Cumulative contribution, %		0.37	0.54		0.40	0.55	

Note:

^a^ Scores in responses of negatively worded questions are reversed.

^b^ The promax rotation was conducted.

^c^ Item Q5 and Q6 were excluded from the final version of the CCS.

The contribution of the factor “socializing” (6 items) was 0.37, that of “belonging” (4 items) was 0.19, and the cumulative contribution was 0.51 in the local volunteers. The contribution of the factor “socializing” (6 items) was 0.40, that of “belonging” (4 items) was 0.15, and the cumulative contribution was 0.55 in the general residents. Item Q5 and Q6 were placed in “belonging” in the tentative CCS, but they were moved to “socializing” in the analysis. In particular, both values of factor loading I and II were close to each other in local volunteers (Q5; factor I=0.56, factor II=0.51) (Q6; factor I=0.67, factor II=0.48). Therefore, the meanings of item Q5 and Q6 could be vague or unstable and we excluded them from the final CCS. The final CCS was shown in Additional file 1: Appendix: Table S4 and Japanese version of that was shown in Additional file 2: Appendix: Table S5.

### Internal consistency and concurrent validity of the final CCS

Mean scores of the final CCS were 17.1(SD 3.7) in local volunteers and 13.5 (SD 4.0) in general residents, with both values of skewness or kurtosis ranging within ± 1 (see Table 3). Regarding internal consistency, Cronbach’s alphas for the CCS were 0.75 for local volunteers and 0.78 for general residents (see Table 3).

**Table 3:** Internal consistency and concurrent validity of the final version of the CCS

		**Local volunteers N=859**	**General residents N=3,484**
**Basic statistics of the CCS (8 items)**	*Mean(SD)*	17.1(3.7)	13.5(4.0)
	*Range*	0-24	0-24
	*Skewness*	−0.27	0.22
	*Kurtosis*	−0.1	0.04
**Internal consistency**			
The CCS (8 items)	*Cronbach’s α*	0.75	0.78
**Concurrent validity**			
BSCS^a^	Correlation, *R*	0.54	0.62
Self- efficacy of helping elderly neighbors^b^			
1) I can call on elderly neighbors, if I do not see them for a few days.	Not at all, *LS means*	13.3	11.0
	Not slightly, *LS means*	15.9	12.4
	Slightly, *LS means*	17.7	14.4
	Absolutely, *LS means*	19.1	16.5
	*η*^*2*^*(95%CI)*	0.13(0.09-0.17)	0.15(0.12-0.17)
2) I can help elderly neighbors with chores such as grocery shopping or garbage disposal.	Not at all, *LS means*	15.2	11.4
	Not slightly, *LS means*	16.3	12.6
	Slightly, *LS means*	17.9	14.2
	Absolutely, *LS means*	20.0	16.4
	*η*^*2*^*(95%CI)*	0.11(0.07-0.15)	0.12(0.10-0.14)

Note:

^a^ Pearson’s correlation.

^b^ Analysis of variance was conducted. Dependent variable was total scores of the final CCS; independent variables were confidence levels of each question of self-efficacy for helping elderly neighbors.

Regarding concurrent validity, the correlation coefficients between the scores of the CCS and BSCS were 0.54 (local volunteers) and 0.62 (general residents) (see Table 3).

Analysis of variance comparing the CCS between the confidence levels of the two types of single question of self-efficacy for helping elderly neighbors showed a large effect size in volunteers (eta squared 0.13; 0.11) and residents (eta squared 0.15; 0.12) (see Table 3). Strong confidence for helping elderly neighbors was associated with high scores on the CCS.

## Discussion

The results of the present study demonstrate that the brief scale that we developed, the CCS to measure CC, showed acceptable internal consistency and concurrent validity among both the local volunteers and the general residents in Japanese urban areas. CC measured by the CCS was related to a similar concept, SOC, and also to self-efficacy for helping elderly neighbors to prevent elderly social isolation, and their concurrent validity was identified.

Our scale development process was solid, as we tested the CCS through population-based surveys of 859 local volunteers as a target sample and 3,484 general residents as a control sample, after pilot testing with 266 local volunteers. One of the strengths of our study is to clarify that the two domains of “belonging” and “socializing” were composed of exactly the same items in both types of sample. The present results suggest that the structure of the CCS is strongly confirmed.

The results also suggest that the statistical evaluation of the CCS was adequate. The distribution including skewness and kurtosis was parametric and the internal consistency was sufficient, as shown by Cronbach’s alphas of 0.75 and 0.78, both achieved at an adequate level for an 8-item scale [31].

Since CC was highly associated with SOC which has been measured in other countries, concurrent validity of the CCS was verified. Compared with the SOC based on McMillan and Chavis [15,16], which includes needs fulfillment, group membership, influence and emotional connection, CC is more focused on mutual relationship between people living in the local community rather than SOC.

The CCS is aiming to measure CC, which relates to helping elderly neighbors in our study perspective. We had assumed that general residents would not actually experience helping elderly neighbors very often. However, the results have shown that local volunteers as well as general residents who are committed to their communities were more confident in both forms of helping elderly neighbors to prevent their social isolation. Previous studies have also shown the relationships between strong SOC and being a volunteer [12,13]. Although relationships between self-efficacy and actual practices of helping elders should be confirmed in future, our results suggest the possibility that enhanced CC among community-dwelling people may facilitate the helping of elderly neighbors and could be measured as an outcome indicator of any intervention for facilitating such helping by utilizing this scale.

The present study has several limitations that suggest caution about generalizing the results. First, response rates of the two surveys, although quite high at about 60%, do not suggest that local volunteers or residents who did not respond to survey have less commitment to their community than the participants who did respond.

Second, the present study setting is limited to Japanese suburban areas close to a megalopolis. People may gather from various local areas in Japan to those cities and have diverse cultures. CC can be especially affected by culture or values in the community and can differ by community characteristics according to whether the area is rural or urban. There may be specific Japanese characteristics in CC compared to other countries. Future studies need to investigate the validity of the CCS in residents living in rural areas in Japan or in other countries, who may have different characteristics from the present participants. Moreover, in using the CCS English version, its validity or reliability should be verified, because the face validity of the CCS was limited.

Third, our study subjects were relatively older residents, but younger people could be local volunteers, so that a cross-generational investigation in CC is needed.

Finally we found evidence regarding the face validity and concurrent validity of the CCS. Other types of concurrent validity or criterion validity including predictive validity or discriminant validity need to be investigated. In particular, the known-group validity of the CCS, for example, according to the groups of local volunteers or general residents, or years of living in the area, should be identified in further analyses.

## Conclusions

We conclude that the Community Commitment Scale composed of 8 items and the two dimensions of “belonging” and “socializing” developed in the present study is a useful confirmed scale showing acceptable internal consistency and concurrent validity. Community commitment measured by the CCS could relate to self-efficacy for helping elderly neighbors, thus preventing their social isolation.

## Abbreviations

BSCS: Brief sense of community scale; CC: Community commitment; CCS: Community commitment scale; SOC: Sense of community.

## Competing interests

The authors have no competing interest to disclose related to the present article.

## Authors’ contributions

All authors contributed to the study design, generating of items, and revision of the scale. AK and YK collected data of the local volunteers in Osaka and ET, YD, WI, and YI collected that of the general residents in Yokohama. AK analyzed and interpreted the data and wrote the manuscript. All authors read and approved the final manuscript.

## Pre-publication history

The pre-publication history for this paper can be accessed here:

http://www.biomedcentral.com/1471-2458/12/903/prepub

## Supplementary Material

Additional file 1 **Appendix: Table S1.** Item Analysis on Pilot Study Participants (N=266). **Appendix: Table S2**. Factor Analysis on Pilot Study Participants (N=266). **Appendix: Table S3**. Distribution, Skewness, and Kurtosis in each Item of the CCS in Study Participants. **Appendix: Table S4**. Community Commitment Scale (CCS) Items. Click here for file

Additional file 2 **Appendix: Table S5.** Community Commitment Scale Japanese version. Click here for file
