# Supplementary material for: Development of a Community Commitment Scale with Cross-sectional Survey Validation for Preventing Social Isolation in Older Japanese People
Source: BMC Public Health. 2012 Oct 24;12:903. doi: 10.1186/1471-2458-12-903 (PMC3533914; doi:10.1186/1471-2458-12-903)
Supplement: Additional file 1 — Appendix: Table S1. Item Analysis on Pilot Study Participants (N=266). Appendix: Table S2. Factor Analysis on Pilot Study Participants (N=266). Appendix: Table S3. Distribution, Skewness, and Kurtosis in each Item of the CCS in Study Participants. Appendix: Table S4. Community Commitment Scale (CCS) Items. [file 1471-2458-12-903-S1.doc]

**Appendix: Table S1. Item Analysis on Pilot Study Participants (N=266)**

|  |  | Difficulty | Distribution^b^ | Item-to-total  correlation |
| --- | --- | --- | --- | --- |
| PQ1^d^ | The elderly should not be isolated in the community. | 1.9% | 98.5% | - |
| PQ2^d^ | I have concern whether the area is easy to live for the elderly. | 4.5% | 79.9% | 0.23 |
| PQ3^ad^ | I feel some difficulty if there is a household having only elderly people nearby. | 3.0% | 84.0% | 0.26 |
| PQ4^d^ | When the elderly have trouble, I’d like them to ask for help. | 1.5% | 98.9% | - |
| PQ5^d^ | I’d like healthy elderly people to take part in local activities. | 1.5% | 96.2% | - |
| PQ6^ad^ | As I get older, I don’t want any help from neighbors. | 2.3% | 52.5% | 0.18 |
| PQ7^d^ | The elderly teach us local customs and wisdom. | 1.9% | 69.1% | 0.44 |
| PQ8^ad^ | It is annoying to listen to elderly people’s talk. | 2.6% | 89.4% | 0.48 |
| PQ9 | I feel a kind of attachment to the community where I live. | 0.8% | 89.8% | 0.57 |
| PQ10^d^ | When people say bad things about my community, I feel they insult me. | 1.1% | 62.1% | 0.25 |
| PQ11 | I want to continue living this community. | 1.1% | 90.9% | 0.49 |
| PQ12^a^ | I don’t feel I am a member of this community. | 2.3% | 89.4% | 0.46 |
| PQ13^ad^ | It is best to leave activities for improving the community to the local people who are enthusiastic about it. | 0% | 86.3% | 0.25 |
| PQ14^a^ | I am hesitant to take part in my neighborhood association activities, because my duties may increase. | 1.5% | 71.1% | 0.50 |
| PQ15 | I feel that my neighborhood association activities are worth doing. | 1.5% | 74.6% | 0.46 |
| PQ16^d^ | I want to contribute as much as possible for the community where I live. | 0.8% | 94.7% | - |
| PQ17^ad^ | It’s good for my neighbors and me to know one another. | 0.8% | 99.6% | - |
| PQ18 | The neighborhood association activities foster friendships among local residents. | 1.5% | 87.5% | 0.59 |
| PQ19^a^ | Socializing in my community is annoying and complicated. | 1.1% | 88.3% | 0.53 |
| PQ20^a^ | I am not interested in my neighbors. | 3.0% | 87.7% | 0.46 |
| PQ21 | My neighbors often greet one another. | 2.3% | 84.4% | 0.40 |
| PQ22 | My neighbors speak regularly and are concerned for one another. | 1.5% | 76.8% | 0.52 |
| PQ23 | I enjoy spending time with my neighbors. | 0.8% | 79.9% | 0.66 |
| PQ24 | My neighbors help me whenever I am in need. | 1.9% | 72.4% | 0.56 |

Note;

^a^ Scores in responses of negatively worded questions are reversed.

^b^ Distribution represents the response percent including “slightly agree” and “strongly agree”.

^c^ Tentative dimensions include: “concerns for the elderly” (PQ1-8), “belonging and contribution”(PQ9-16), and “cohesion and socializing” (PQ17-24).

^d^ Items (a total of 12 items) were excluded for the exploratory factor analysis.

**Appendix: Table S2. Factor Analysis on Pilot Study Participants (N=266)**

|  |  | Factor Loading | |
| --- | --- | --- | --- |
|  |  | I | II |
| PQ14 | I am hesitant to take part in my neighborhood association activities, because my duties may increase. | .64 | .19 |
| PQ9 | I feel a kind of attachment to the community where I live. | .71 | .37 |
| PQ12 | I don’t feel I am a member of this community. | .63 | .22 |
| PQ20 | I am not interested in my neighbors. | .60 | .18 |
| PQ19 | Socializing in my community is annoying and complicated. | .66 | .39 |
| PQ11 | I want to continue living this community. | .64 | .37 |
| PQ15 | I feel that my neighborhood association activities are worth doing. | .60 | .28 |
| PQ18 | My neighborhood association activities foster friendships among local residents. | .61 | .58 |
| PQ22 | My neighbors speak regularly and are concerned for one another. | .31 | .89 |
| PQ21 | My neighbors often greet one another. | .22 | .80 |
| PQ24 | My neighbors help me whenever I am in need. | .40 | .80 |
| PQ23 | I enjoy spending time with my neighbors. | .58 | .74 |
| Contribution | | .38 | .13 |
| Cumulative contribution | | .38 | .51 |

The promax rotation was conducted.

**Appendix: Table S3. Distribution, Skewness, and Kurtosis in each Item of the CCS in Study Participants**

|  | | Local volunteers N=859 | | | General residents N=3484 | | |
| --- | --- | --- | --- | --- | --- | --- | --- |
|  |  | Distribution | Skewness | Kurtosis | Distribution | Skewness | Kurtosis |
| *Belonging* | |  |  |  |  |  |  |
| Q1^c^ | I feel a kind of attachment to the community where I live. | 95.3% | 1.03 | 0.84 | 82.7% | 0.76 | 0.30 |
| Q2^c^ | I want to continue living this community. | 93.6% | 1.30 | 1.52 | 86.8% | 0.74 | 0.53 |
| Q3^a^ | I don’t feel I am a member of this community. | 80.0% | 1.09 | 0.08 | 64.8% | 0.90 | 0.80 |
| Q4^a^ | I am hesitant to take part in my neighborhood association activities,  because my duties might increase. | 63.4% | 0.25 | 0.77 | 43.3% | 0.88 | 0.77 |
| Q5 | I feel that my neighborhood association activities are worth doing. | 77.8% | 0.39 | 0.08 | 38.8% | 0.79 | 0.63 |
| Q6 | The neighborhood association activities foster friendships  among local residents. | 85.4% | 0.67 | 0.10 | 66.4% | 0.79 | 0.33 |
| Q7^a^ | Socializing in my community is annoying and complicated. | 85.7% | 0.73 | 0.15 | 61.6% | 0.78 | 0.48 |
| Q8^a^ | I am not interested in my neighbors. | 80.5% | 0.73 | 0.01 | 61.2% | 0.08 | 0.45 |
| *Socializing* | |  |  |  |  |  |  |
| Q9 | My neighbors often greet one another. | 90.6% | 0.75 | 0.37 | 83.2% | 0.57 | 0.04 |
| Q10 | My neighbors speak regularly and are concerned for one another. | 85.1% | 0.46 | 0.13 | 61.6% | 0.09 | 0.64 |
| Q11 | I enjoy spending time with my neighbors. | 84.7% | 0.40 | 0.07 | 44.7% | 0.18 | 0.49 |
| Q12 | My neighbors help me whenever I am in need. | 82.4% | 0.30 | 0.02 | 54.3% | 0.09 | 0.46 |

^a^ Scores in responses of negatively worded questions are reversed.

^b^ Distribution represents the response percent including “slightly agree” and “strongly agree”.

^c^ Item Q1 and Q2 were excluded for the CFA.

**Appendix: Table S4. Community Commitment Scale (CCS) Items**

Please choose the rating closest to your thought for each statement.

|  | | *Not confident at all* | *Not confident slightly* | *Confident slightly* | *Absolutely confident* |
| --- | --- | --- | --- | --- | --- |
| *Socializing* | |  |  |  |  |
| 1. | My neighbors speak regularly and are concerned for one another. | 0 | 1 | 2 | 3 |
| 2. | My neighbors help me whenever I am in need. | 0 | 1 | 2 | 3 |
| 3. | I enjoy spending time with my neighbors. | 0 | 1 | 2 | 3 |
| 4. | My neighbors often greet one another | 0 | 1 | 2 | 3 |
| *Belonging* | |  |  |  |  |
| 5.^a^ | Socializing in my community is annoying and complicated. | 0 | 1 | 2 | 3 |
| 6.^a^ | I am not interested in my neighbors. | 0 | 1 | 2 | 3 |
| 7.^a^ | I am hesitant to take part in my neighborhood association activities, because my duties might increase. | 0 | 1 | 2 | 3 |
| 8.^a^ | I don’t feel I am a member of this community. | 0 | 1 | 2 | 3 |

^a^ Scores in responses of negatively worded questions need to be reversed.
