# Supplementary material for: Development of a Community Commitment Scale with Cross-sectional Survey Validation for Preventing Social Isolation in Older Japanese People
Source: BMC Public Health. 2012 Oct 24;12:903. doi: 10.1186/1471-2458-12-903 (PMC3533914; doi:10.1186/1471-2458-12-903)
Supplement: Additional file 2 — Appendix: Table S5. Community Commitment Scale Japanese version. [file 1471-2458-12-903-S2.pdf]

Appendix: Table5. Community Commitment Scale Japanese version

地域コミットメント尺度　日本語版

あなたの「地域での人付き合いや活動」に対するお考えを伺います。
各項目についてあなたの考えに最も近いと思う番号に〇を1つつけて下さい。

	全く<br>思わない	あまり<br>思わない	やや<br>そう思う	とても<br>そう思う
つきあい				
1. この地域の人々は日頃から互いに気づかったり、<br>声をかけ合っている	0	1	2	3
2. この地域の人々は困った時は助けてくれる	0	1	2	3
3. 地域の人たちと過ごす時間は楽しい	0	1	2	3
4. この地域の人々はよくあいさつをしている	0	1	2	3
帰属感				
5. 地域での人づきあいはわずらわしい	0	1	2	3
6. 地域の人のことについては関心がない	0	1	2	3
7. 町内会（自治会）などに行くと役割が増えそうで気が重い	0	1	2	3
8. 私はこの地域の一員とは感じられない	0	1	2	3
